# Biomarkers and gene copy number variation for terpenoid traits associated with insect resistance in Sitka spruce: An integrated genomic, proteomic, and biochemical analysis of (+)-3-carene biosynthesis

**DOI:** 10.1186/1753-6561-5-S7-O27

**Published:** 2011-09-13

**Authors:** Joerg Bohlmann, Dawn Hall, Jeanne Robert, Christopher Keeling, Dominik Domanski, Alfonso Lara Quesada, Sharon Jancsik, Michael Kuzyk, Britta Hamberger, Christoph Borchers

**Affiliations:** 1 Michael Smith Laboratories, University of British Columbia, Canada; 2 University of Victoria – Genome BC Proteomics Centre, Canada; 3 University of Victoria – Genome BC Proteomics Centre and Department of Biochemistry, University of Victoria, Canada

## 

Conifers have evolved complex chemical defenses in the form of oleoresin terpenoids to resist attack from pathogens and herbivores. The large diversity of terpenoid metabolites is determined by the size and composition of the terpene synthase (TPS) gene family, and the single- and multi-product profiles of these enzymes. The monoterpene (+)-3-carene is associated with resistance of Sitka spruce (*Picea sitchensis*) to white pine weevil (*Pissodes strobi*). We used a combined genomic, proteomic and biochemical approach to analyze the (+)-3-carene phenotype in two contrasting Sitka spruce genotypes. Resistant trees produced significantly higher levels of (+)-3-carene than susceptible trees, in which only trace amounts were detected. Biosynthesis of (+)-3-carene is controlled, at the genome level, by a small family of closely related (82-95% amino acid sequence identity) (+)-3-carene synthase (*PsTPS-3car*) genes. Transcript profiling identified one *PsTPS-3car* gene (*PsTPS-3car1*) which is expressed in both genotypes, one gene (*PsTPS-3car2*) expressed only in resistant trees, and one gene (*PsTPS-3car3*) expressed only in susceptible trees. The *PsTPS-3car2* gene was not detected in genomic DNA of susceptible trees. Target-specific selected reaction monitoring substantiated this pattern of differential expression of members of the PsTPS-3car family on the proteome level. Kinetic characterization of the recombinant PsTPS-3car enzymes identified differences in the activities of PsTPS-3car2 and PsTPS-3car3as a factor for the different (+)-3-carene profiles of resistant and susceptible trees. In conclusion, variation of the (+)-3-carene phenotype is controlled by *PsTPS-3car* gene copy number variation, variation of gene and protein expression, and variation of catalytic efficiencies.
